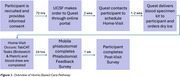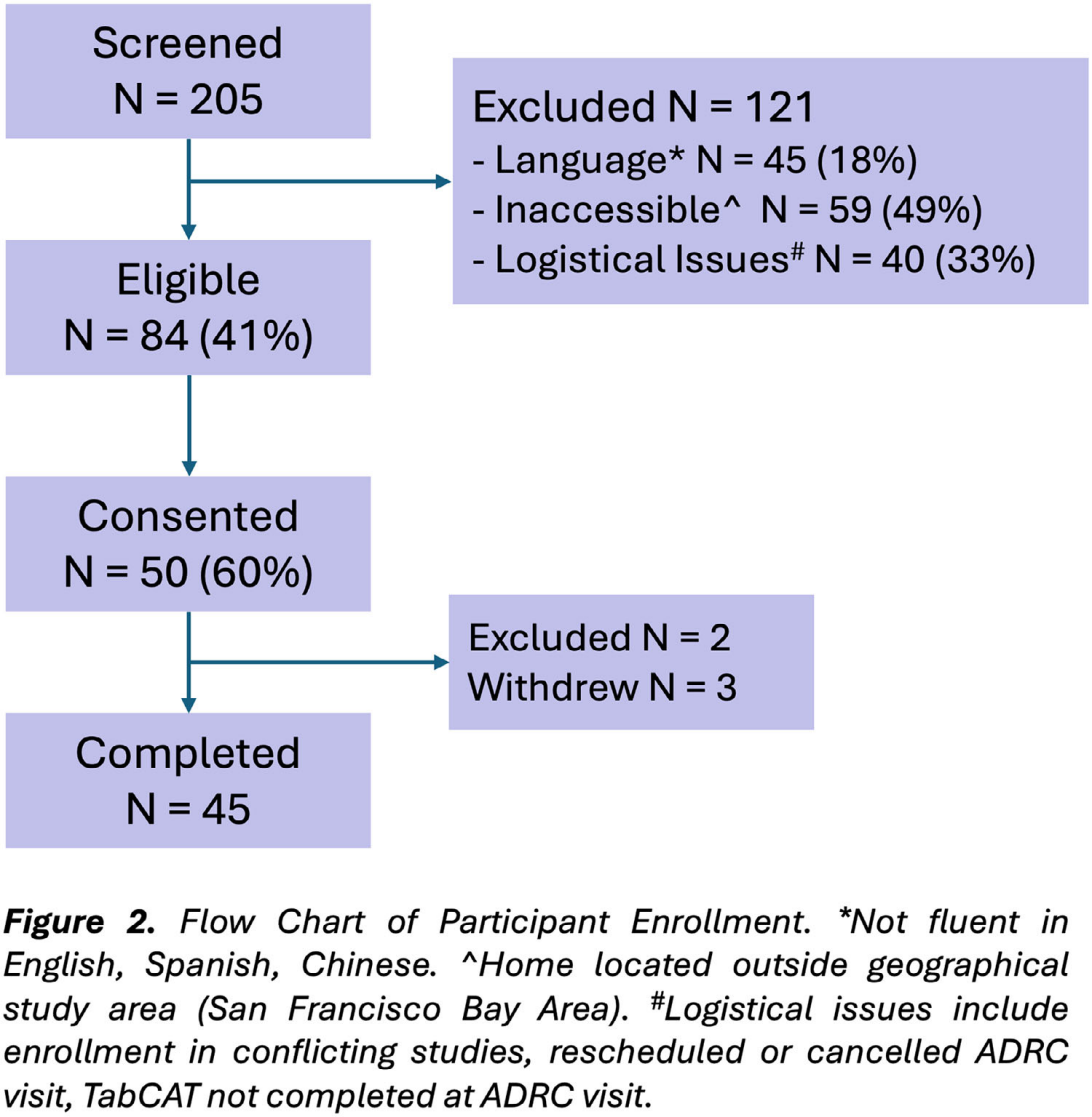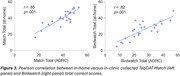# Evaluation of a Novel Home‐Based Pathway to Support Early Detection and Diagnosis of Alzheimer's Disease

**DOI:** 10.1002/alz70857_103571

**Published:** 2025-12-25

**Authors:** Kelly J. Atkins, Tiffany R. Brailow, Alexandra J. Weigand, Elena Tsoy, Michael K Racke, Julia Larsen, Claudio Reck‐Rivera, Sabrina J Erlhoff, Argentina Lario Lago, Charles C. Windon, Peggye Dilworth‐Anderson, Katherine L. Possin

**Affiliations:** ^1^ Memory and Aging Center, University of California San Francisco, San Francisco, CA, USA; ^2^ University of California San Francisco, San Francisco, CA, USA; ^3^ Global Brain Health Institute, University of California, San Francisco, San Francisco, CA, USA; ^4^ Quest Diagnostics Nichols Institute, San Juan Capistrano, CA, USA; ^5^ University of California, San Francisco, San Francisco, CA, USA; ^6^ Department of Neurology, University of California, San Francisco, San Francisco, CA, USA; ^7^ University of North Carolina, Chapel Hill, Chapel Hill, NC, USA

## Abstract

**Background:**

Delays in the diagnosis of Alzheimer's disease and related dementias (ADRD) impede access to disease‐modifying interventions, supportive care, and the opportunity to participate in research. We examined the feasibility, acceptability, reliability, and validity of a novel home‐based diagnostic pathway that leverages advances in digital cognitive assessments and plasma biomarkers.

**Method:**

Ethnically diverse participants from the UCSF Alzheimer's Disease Research Center (ADRC) were recruited and completed in‐home digital cognitive assessment (TabCAT Brain Health Assessment) and blood biospecimen collection with a Quest mobile phlebotomist, as well as parallel procedures in the ADRC. Participants completed testing in their preferred language (22 English, 19 Spanish, and four Chinese). Feasibility and acceptability were evaluated using phlebotomist and participant feedback following the encounter. We evaluated the reliability and validity of the TabCAT‐BHA and AD plasma biomarkers (pTau‐217, NfL, GFAP, Aβ42/40) collected in the home against data collected in‐clinic by the ADRC (Figure 2).

**Result:**

Forty‐five participants completed the pathway (Mean[SD]_age_=69.2[9.7]; 69% female), including 24 who were cognitively unimpaired, 14 with MCI, and seven with dementia (Figure 1). The home pathway was feasible and acceptable for all 35 phlebotomists, who reported no challenges in 79% of encounters and minor challenges in 21%. Eighty percent of participants reported a preference for home‐based assessment compared to in‐clinic. Test‐retest reliability of the TabCAT subtests across settings was excellent (Match, *r* = 0.85, *p* < 0.001; Birdwatch *r* = 0.82, *p* < 0.001; Figure 3). The ROC analysis for in‐home TabCAT tasks after adjusting for sex, age, and education yielded an area under the curve (AUC) of 0.92 for separating cognitively impaired (i.e., MCI or dementia) from unimpaired, (95% CI: 0.81–0.99), with sensitivity of 0.85 and specificity of 0.95. At the time of this submission, plasma biomarker measurements are underway, and the results will be reported by July 2025.

**Conclusion:**

This novel home‐based pathway is a promising approach for the collection of digital cognitive assessments and ADRD plasma biomarkers, potentially aiding in early diagnosis and informing disease modifying treatment eligibility. Importantly, this pathway may also reduce barriers to testing and expanding access to dementia care.